# Normal Retinotopy in Primary Visual Cortex in a Congenital Complete Unilateral Lesion of Lateral Geniculate Nucleus in Human: A Case Study

**DOI:** 10.3390/ijms23031055

**Published:** 2022-01-19

**Authors:** Akshatha Bhat, Jan W. Kurzawski, Giovanni Anobile, Francesca Tinelli, Laura Biagi, Maria Concetta Morrone

**Affiliations:** 1 IRCCS Stella Maris, 56128 Pisa, Italy; akshathabhat8@gmail.com (A.B.); jan.kurzawski@gmail.com (J.W.K.); giovannianobile@hotmail.it (G.A.); f.tinelli@fsm.unipi.it (F.T.); laura.biagi@fsm.unipi.it (L.B.); 2 Department of Neuroscience, University of Florence, 50121 Firenze, Italy; 3 Department of Neuroscience, Psychology, Pharmacology, and Child Health, University of Florence, 50139 Florence, Italy; 4 Department of Translational Research in Medicine, University of Pisa, 56126 Pisa, Italy

**Keywords:** microphthalmia, visual plasticity, population receptive field mapping, BOLD retinotopy, tractography of visual pathways

## Abstract

Impairment of the geniculostriate pathway results in scotomas in the corresponding part of the visual field. Here, we present a case of patient IB with left eye microphthalmia and with lesions in most of the left geniculostriate pathway, including the Lateral Geniculate Nucleus (LGN). Despite the severe lesions, the patient has a very narrow scotoma in the peripheral part of the lower-right-hemifield only (beyond 15° of eccentricity) and complete visual field representation in the primary visual cortex. Population receptive field mapping (pRF) of the patient’s visual field reveals orderly eccentricity maps together with contralateral activation in both hemispheres. With diffusion tractography, we revealed connections between superior colliculus (SC) and cortical structures in the hemisphere affected by the lesions, which could mediate the retinotopic reorganization at the cortical level. Our results indicate an astonishing case for the flexibility of the developing retinotopic maps where the contralateral thalamus receives fibers from both the nasal and temporal retinae.

## 1. Introduction

Retinotopic representations of the visual field are a principal feature of the visual system, which is the outcome of the delicate interplay between preprogrammed and experience-dependent mechanisms during early development [1,2]. The connections between the sensory organs and the brain are critical in preserving topographic maps. The decussation of the optic nerve at Optic chiasm (OC) is essential in achieving the contralateral representation of the visual field in each hemisphere; however, this can be altered in many pathologies [3] such as albinism [4,5] or FHONDA syndrome [6].

These pathologies have genetic origin and can induce alteration in the development of the visual pathway during very early embryonic stage. However, congenital lesions have also demonstrated the induction of miss-crossing of the optic tract, specifically when the lesion takes place before the time the retinal ganglion cell axons reach the OC (about 48 days of gestation). The age at which the lesion is acquired (during embryonic, peri-natal, or post-natal phases) is a crucial factor in determining the degree of rerouting and reorganization of the developing visual pathways and visual function [7].

Acquired cortical damage of the adult visual system can result in complete loss of visual input to early visual areas, often resulting in scotomas in the corresponding visual field or in complete blindness [7,8,9]. There is still a lack of unequivocal account for the mechanism of the subsequent reorganization in the adult visual system [10], but the reorganization of cortical damage at an early age is well documented. 

A large study of subjects with occipital cortical lesions, where the damage occurred within late teens and around 30 years, reported correlation between age at lesion and the probability that the scotoma shrinks during the years succeeding brain injury [11]. Comparably, the recovery of visual capabilities was greater in patients who underwent hemispherectomy at the age of 7 years as compared to cases where the surgery occurred later in life [12]. In clinical cases, where the cortical damage is prenatal or in early infancy, the developing visual system can display a considerable degree of adaptive plasticity, even several years after the occurrence of a lesion during childhood [13]. Similarly, Werth (2006) [14] reported the case of a child who underwent hemispherectomy at 4 months of age but later developed a normal visual field comparable to age-matched controls, confirming previous evidence of functional responses in the blind field of hemispherectomy infants [15].

A large literature documents a great re-routing of visual pathways, as well as in somatosensory and motor pathways, but only in the presence of congenital damage [16,17]. Muckli et al. [18] reported visual field maps for an individual who lost large parts of the right hemisphere, including the LGN and optic nerve, during embryonic development. This condition was also associated with microphthalmia of the right eye. They found that the left optic nerve projects entirely ipsilateral to the left hemisphere [18]. This resulted in bilateral visual field maps with an interlaced cortical retinotopy.

In the present study, we measured the residual perceptual capacities of a 12-year-old girl IB with a congenital and extensive brain lesion in the left hemisphere along with a microphthalmos left eye. Microphthalmia is a rare condition (combined birth prevalence: up to 30 per 100,000 cases; [19]) that is associated with an absence and reduction of eye size in the orbit, with normal adnexal elements and eyelids usually present. In severe monocular microphthalmia, the lateralization of the optic nerve projections of the fellow eye was reported to be normal [20,21]. However, in the case of Patient IB, the contralateral LGN (left) is missing, and the thalamic projections from the fellow eye (right eye) need to be re-organized. 

Despite lacking inputs to the affected side of the striate cortex, IB has surprisingly sparse scotoma, suggesting the occurrence of a dramatic reorganization of visual pathways. To understand her good visual capabilities, we measured the contrast sensitivity and retinotopic organization of the patient along with white matter tractography. We revealed a profound rerouting of the visual information necessary to achieve a normal cortical retinotopic representation in the primary visual cortex.

## 2. Results

### 2.1. Behavioral Results

The patient IB’s visual field was evaluated by an automated perimetry system (KOWA AP 340: similar to the Humphrey perimeter). IB’s left eye has microphtalmia; hence, it cannot detect any light. The right eye was tested for 283 different locations, covering the full field of view of 120 × 120 degrees. The central fixation was monitored throughout the session while presenting light targets of varied luminance. The visual field of Patient IB (Figure 5D) is largely spared and clearly covers the contra-lesioned visual field (right), with a very sparse scotoma in the lower right visual field only, after 15° of eccentricity. 

**Figure 1:**
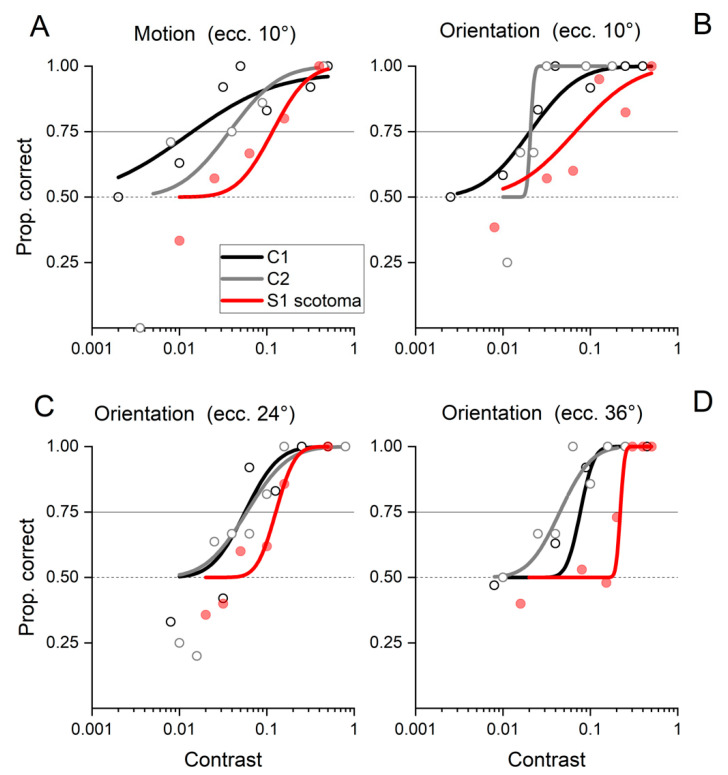
Psychophysical assessment of contrast sensitivity in Patient IB and two age-matched controls. (**A**) Contrast sensitivity for motion discrimination at 10° of horizontal eccentricity. (**B**–**D**) Contrast sensitivity for orientation discrimination at 10°, 24°, and 36° of horizontal eccentricity, respectively.

In addition, the contrast sensitivity measured with two different tasks (motion discrimination and orientation discrimination) for Patient IB is comparable to those obtained with two age-matched controls (see methods for details). Patient IB had thresholds for motion discrimination (Figure 1A) and orientation discrimination (Figure 1B) at 10° of eccentricity around 9% contrast, while the threshold was around 2% for two age-matched controls (C1 and C2 threshold). Then, we further tested for orientation discrimination at 24° and 36° of horizontal eccentricity (Figure 1C,D). Patient IB had thresholds of 12% and 22% for 24° and 36° of eccentricities, while the age-matched controls had thresholds around 5% for 24° and 7% for 36° of eccentricity. It is important to note that Patient IB always performed above chance in all the eccentricities for high-contrast stimuli. This remarkably good visibility contrasts with the massive damage in the thalamic visual pathways and hints at the possibility of some compensatory mechanisms in action to compensate for the absence of direct optic radiation innervating V1 in the lesioned hemisphere.

### 2.2. PRF Mapping Reveals a Retinotopic Map of the Visual Cortex

**Figure 2:**
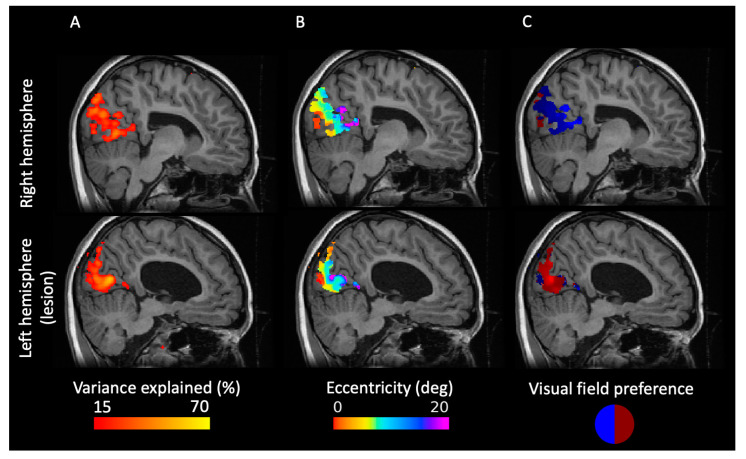
Visual cortex of patient IB is retinotopically organized. (**A**) Map of variance explained estimated from pRF mapping. The higher the value, the more confident the estimation of the pRF. (**B**) Eccentricity map shows an orderly representation of the visual field in both hemispheres. (**C**) Preference of visual field location along the x-axis. Each hemisphere processes the information from the contralateral visual field. All maps are masked with the variance-explained threshold of 15%.

To assess the retinotopic organization of the visual cortex, we employed the model-driven approach of pRF mapping. Since the behavioral results showed that patient IB has only a sparse and diffuse scotoma in the lower right visual field and intact early visual cortex in both hemispheres, we hypothesized an orderly organized retinotopic map. In both hemispheres, we find a good fit of the retinotopic model (Figure 2A) with the central visual field represented close to the occipital pole and peripheral visual field represented anterior from the occipital lobe (Figure 2B). The eccentricity map extends to locations up to the POS, revealing a good fit of the pRF maps that possibly include the peripheral area prostriata [22]. e also find that each hemisphere processes the information from the contralateral visual field (Figure 2C). Our findings show that the retinotopic organization of IB’s visual cortex is similar to that found in healthy subjects [23]. However, this does not reveal the origin of the signal, as the thalamus in the affected hemisphere is entirely missing. 

### 2.3. Reorganization of Responses in the Thalamus

**Figure 3:**
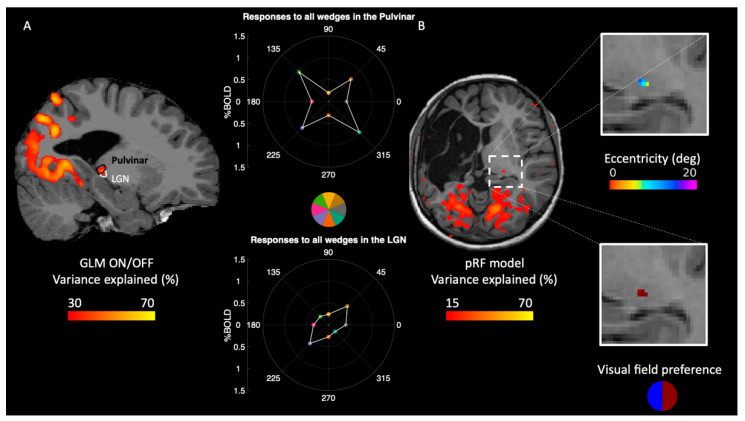
GLM results in right thalamus. (**A**) Variance explained by the GLM (all wedges vs. blank) plotted on one slice of the anatomical brain. Black and white contours plot the LGN and pulvinar extracted from the atlas. The inset shows responses to each of the wedges extracted from both ROIs. (**B**) pRF mapping of the thalamus showing the eccentricity and visual field preference maps. pRF maps are thresholded with the variance-explained threshold of 15%.

In normal subjects, the major input to V1 is from LGN via optic radiation that carries information from the contralateral visual field. However, due to the lesion of the thalamus and of the optic radiation in the left hemisphere, the right thalamus might be involved in processing the ipsilateral visual field inputs. Contrasting all wedge stimuli versus blank, we locate a reliable response within the intact thalamus (Figure 3A; outlined in dashed white and black line). Figure 3A shows that the both the LGN and the pulvinar respond well to stimuli centered in the eight visual field locations (three wedges located in the left, three located in the right hemifield, and two located at the cardinal meridians), suggesting that they represent both the ipsi-lateral and the contra-lateral visual field signal. Both ROIs were taken from a recently released atlas of the thalamic nuclei [24] and projected on the right hemisphere of IB. Finally, pRF maps revealed an organized retinotopic map in the activated thalamic region with an ipsilateral visual field preference (Figure 3B). Note that both the GLM and pRF mapping vary in methodological approach, yet they both point to the same subcortical region. In patient IB, it is challenging to anatomically segregate the pulvinar from LGN due to the difficulty in estimating the impact of the deformation of the lesioned hemisphere on the intact hemisphere. Given the more medial localization of the activity and that foveal–peripheral retinotopic map is similar to previous reports [25], it is mostly likely that the foci in Figure 3B is part of visual pulvinar. 

### 2.4. Visual White Matter Bundles in Patient IB

**Figure 4:**
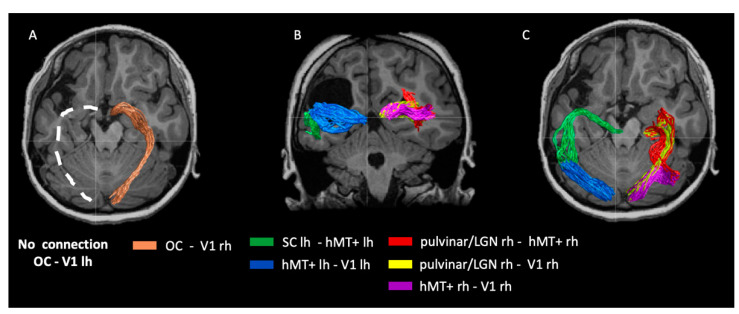
Diffusion tractography in patient IB. (**A**) OC connects with V1 only in the intact hemisphere. (**B,C**) Importantly, a connection between the SC and hMT+ that extends to V1 is found in the lesioned hemisphere. Visual white matter bundles in the intact and healthy hemisphere shown in the coronal (**B**) and axial (**C**) views. Rh and lh indicate the location of the ROI in the right or in the left hemisphere respectively.

In the previous paragraph, we show that the thalamus in the intact hemisphere responds to both the ipsi-lateral and contra-lateral visual fields. The retinotopic information of the ipsi-lesional visual field might reach V1 in the lesioned hemisphere through the thalamus (considering its full field representation), crossing at the callosal level. Another possibility is that the signal reaches V1 in the lesioned hemisphere directly from the OC and SC. These questions cannot be answered with functional data and need a structural assessment of the white matter. To address these questions, we used diffusion tractography, which allows the mapping of white matter connections between the brain regions. First, we found that OC is connected with V1 only in the intact hemisphere, and this connection is missing in the lesioned hemisphere (Figure 4A). Second, we found a connection between the SC in the lesioned hemisphere (the SC is spared in the lesioned hemisphere) and hMT+ that extends to V1 (Figure 4B,C). Third, we assessed connectivity in the healthy hemisphere and found strong connections between the thalamus and V1, thalamus and hMT+, and V1 with hMT+ (Figure 4B,C). Note that the thalamic ROI includes both the LGN and the pulvinar, as labeled in Figure 3A. In principle, the more dorsal track to hMT+ may originate from the pulvinar, but we do not have the resolution to distinguish between these two alternatives.

Overall, these results show that retinotopic signal from the contralateral lesioned visual field does reach V1 through a reorganization of the thalamus and tectum in the intact hemisphere. A possible pathway may be the retina, thalamus, and SC of the intact hemisphere that crosses the information to the lesioned SC and finally projects to V1 of the lesioned hemisphere directly or through hMT+.

## 3. Methods

### 3.1. Clinical Description

**Figure 5:**
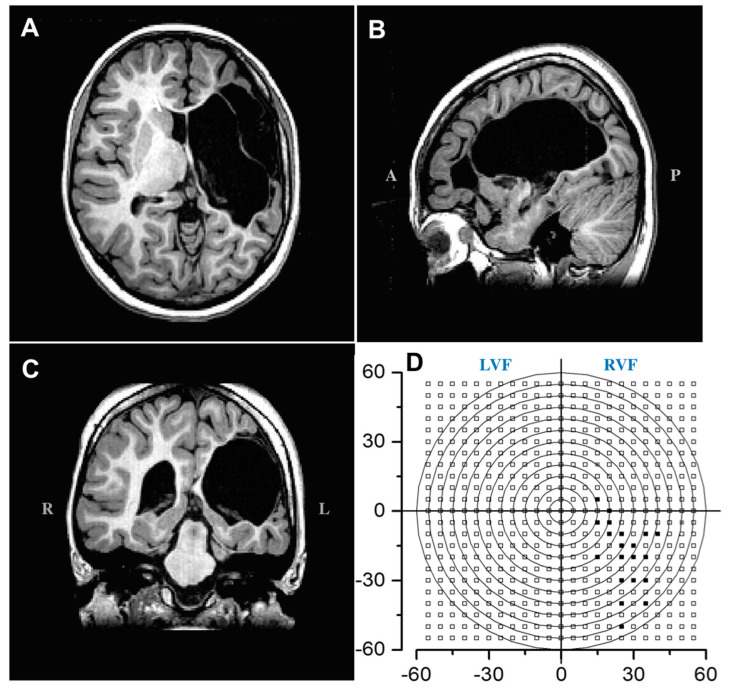
Structural T1-weighted MRI scan and visual field mapping of Patient IB. Sections showing the absence of LGN and optic radiations in the left hemisphere are displayed in (**A**) transverse, (**B**) sagittal (A-Anterior, P- Posterior), and (**C**) coronal planes (R- Right, L- Left). (**D**) Graphical reconstruction of visual field perimetry up to 60° of eccentricity. Visual field perimetry obtained with the KOWA AP 340, retaining 5° resolution of the perimetry. The contra-lateral right visual field has a very sparse scotoma in the lower field only after 15° of eccentricity. Open squares report successful detection at 15 and 17 dBs luminance levels; filled squares show an absence of detection at 0 dBs.

Subject IB is a 12-year-old girl. She was born with a very extensive congenital brain lesion involving half of the left cortical hemisphere (parietal and occipital cortex and partially optic radiations and large portion of the thalamus; Figure 5A–C show a T1-weighted MRI image of the patient after the surgery performed at age 11). She also has microphthalmia and retinal detachment in her left eye, causing the complete loss of vision. During the first year after birth, she had epileptic seizures, which needed medication. IB also has a right hemiplegia involving particularly the upper limb. She is also slow in completing visuo-motor tasks.

IB’s cognitive level, assessed by WISC–IV, was in the range of mild disability (QIT 66) with borderline performance in verbal comprehension (Index 82). While a specific expressive–receptive language disorder is still present, she is now able to read and write. IB’s perceptual reasoning index was 78, processing speed index was 65, and working memory index was 60.

A complete ophthalmological evaluation revealed no refractive error in her right eye. Her visual acuity was 0.9, which is in the normal range. Stereopsis was absent, while color recognition was good. The visual field was assessed by an automated perimetry system (KOWA AP 340) in which luminance detection was tested for each eye for target locations spanning an area of 120 × 120°of the visual field, showing a reduced sensitivity only in the periphery of the lower-right hemifield (beyond 15° of eccentricity). The perimetry test used the normal sensitivity curve evaluated in the preliminary phase of the test, with the background luminance of 31.5 Asb, and targets of 3 different luminance levels, respectively 67 Asb (17 dB), 215 Asb (12 dB), and 3400 Asb (0 dB).

OKN was elicited with black and white stripes moving temporally-to-nasally (TN) or nasally-to-temporally (NT) at velocities of 15, 30, 45, and 60°/s. A strong asymmetry of optokinetic responses favoring the direction toward the hemianoptic visual field was present.

### 3.2. Psychophysical Tests

The contrast sensitivity of IB and two age-matched controls were psychophysically tested. Stimuli for all the psychophysical tests were generated by MATLAB R2019b Available online: www.mathworks.com, (accessed on 4 January 2021) [26] and presented with a refresh rate of 120 Hz on a Pioneer color plasma monitor subtending 80° × 60° at a viewing distance of 57 cm. Stimuli were presented on a gray background. Subject IB was instructed to keep fixation on a central red disk (0.5°). Eye movements were recorded throughout the sessions.

Contrast sensitivity for motion was measured with sinusoidal gratings (1 cpd) that drifted at a temporal frequency of 4 Hz, were framed in a circular aperture of 4°, and presented for 200 ms on either side of the visual field at a horizontal eccentricity of 10°. IB was asked to indicate (verbally) the gratings motion direction (either left or right). The proportion of correct response as a function of grating contrast was fitted by psychometric curves to evaluate the contrast threshold (75% of correct responses). Contrast threshold for static stimuli was also tested by an orientation discrimination task. The grating stimuli parameters were identical to those used for the motion task, but here, they were static and had varied orientation in ±45° angle. The subject indicated (verbally) the perceived orientation. Three different horizontal eccentricities were tested in separate sessions (10°, 24°, and 36°). 

### 3.3. Imaging Methods

#### 3.3.1. Data Acquisition 

Imaging data were acquired on a GE 1.5 T HD Neuro-optimized System (General Electric Medical Systems, Wauwatosa, WI, USA) fitted with 40 mT/m high-speed gradients. The session consisted of one structural and six functional sessions. A whole-brain fast spoiled gradient recalled acquisition in the steady-state T1-weighted series (FSPGR) was collected in the axial plane with TR 10.2 ms, TE 2.4 ms, inversion time (T1) 700 m s, flip angle 1⁄4 10°, yielding 134 continuous 1 mm axial slices with an in-plane resolution of 0.75 mm.

Functional data were acquired with a single-shot gradient-echo, echo planar imaging (EPI) sequence. The acquisition parameters were as follows: 38 axial slices of 3 mm thickness, 64 × 64 matrix, 3 × 3 mm in-plane resolution, 50 m sec echo time (TE), 3000 m sec repetition time (TR), 90° flip angle. The first four volumes of each session were discarded to allow stabilization of the BOLD signal. The coverage included supra-tentorial structures and most of the cerebellum. 

Diffusion data were acquired during the same visit. It consisted of a dataset with b0 image acquired in the beginning of the sequence and 60 equally distributed diffusion directions with bvalue = 3000 s/mm^2^. Initially, the resolution of the diffusion data was 0.75 mm in plane with a slice thickness of 3 mm; however, for the purpose of tractography, it was resampled to 2 mm^3^ isotropic. 

#### 3.3.2. Visual Stimulation for pRF Mapping

The stimuli for all functional magnetic resonance imaging (fMRI) were displayed through liquid crystal goggles (VisuaStim XGA Resonance Technology at a resolution of 800 × 600 voxels, subtending 30° × 22.5° at an apparent distance of 1.5 m, with mean luminance of 30 cd/m^2^). 

We performed scans in four different sessions in order to construct pRF maps. Stimuli were presented binocularly. They were defined as apertures of a mid-level gray mask that uncovered a checkerboard pattern, rotating and contracting at a rate of one check per second. The first session consisted of apertures defined by two 45° wedges centered around 0° or around 90° (we further refer to it as the meridian session). The horizontal and vertical meridian were presented interchangeably for 4 TRs each (without blanks), and the sequence was repeated 6 times for a total of 40 TRs. Additionally, we acquired two separate sessions with the same 45° wedge presented in eight slices covering a 360° visual field. Each wedge lasted about 12 TRs, along with a blank after four consecutive wedges. This repeated twice yielded 160 TRs in total. The last session consisted of rings that partitioned the screen space into three contiguous eccentricity bands (0.5° to 1.5°, 1.5° to 6°, and 6° to 20°). In one run, the three selected rings and one blank were presented for 4 TRs each, with a total of 80 TRs. Stimuli were generated using Psychtoolbox [26] using MATLAB. Eye movements were measured during each scanning session (with Resonance Technology infra-red camera and Arlington Research software, Wilsonville, AZ, USA). No breaks of fixation were observed other than small saccades less than 1°.

### 3.4. Functional Data Analysis

Prior to statistical analysis, functional data underwent pre-processing steps including slice time correction (AFNI’s *3dTshift*) and 3D motion correction (AFNI’s. *3dvolreg* [27]); Functional data were co-registered to the 3D anatomical T1-weighted image by using Pearson’s correlation with the standard 6 degrees of freedom approach using AFNI’s *3dAllineate* [28]. BOLD responses were analyzed using GLMdenoise [29] after the alignment to the anatomical space. A General Linear Model (GLM) was performed as follows. Predicted BOLD time series were modeled by convolving the stimulus onset with a hemodynamic response function that was optimized as a part of GLMdenoise functionality. Finally, to estimate the beta weights, we solved the linear model for each BOLD time course. The design matrix included predicted time series and modeled noise achieved through GLMdenoise functionality.

#### 3.4.1. pRF Mapping 

We used vistasoft. Available online: https://vistalab.stanford.edu/software/ (accessed on 26 November 2021) for pRF estimation, which uses the two-dimensional Gaussian pRF model of Doumulin et al. [30].

$$g\left(x,y\right)=e^{-\left(\frac{{\left(x-x_{o}\right)}^{2}+{\left(y-y_{o}\right)}^{2}}{2\sigma^{2}}\right)}$$

where *x* and *y* define the center of the pRF in the visual field and *σ* is the radius. To estimate the pRF model, we included all retinotopic acquisitions (2 runs of wedges, 1 run of rings, and 1 run of meridians). To find the best fitted 2D Gaussian (parametrized at each voxel), we first created a predicted time-series by multiplying the 2D Gaussian by the stimulus aperture and convolving with an HRF. The HRF describes the difference of two gamma functions and was previously used to successfully predict the retinotopic maps [30,31,32,33,34,35]. For each voxel, we found the best fitted (estimated by variance explained) predicted time course and saved the *x*, *y*, and *σ* of the 2D Gaussian that was used to model it. Using these coordinates, for each voxel, we calculated the eccentricity and estimated the visual field preference.

#### 3.4.2. Analysis of Diffusion Data

Diffusion data were preprocessed and analyzed using vistasoft. Available online: https://vistalab.stanford.edu/software/ (accessed on 26 November 2021). Mean b0 image was registered to T1-weighted anatomy allowing for the creation of a full ‘dt6′ dataset that included diffusion data, b values, b vectors, a T1-image and diffusion metrics. A diffusion tensor was estimated using the Constrained Spherical Deconvolution (CSD) model [36] with maximum harmonic order (lmax) estimated by mrTrix 0.2 [37].

Regions of interest (ROIs) included in the tractography experiment were extracted from functional maps. Definition of the boundaries was either based on pRF mapping (V1), GLM estimates (human middle temporal complex hMT+), anatomical landmarks (OC, SC), or an atlas (right thalamus; Pulvinar + LGN). We included the following ROIs: OC, left superior colliculus, right SC, left hMT+, right hMT+, left V1, right V1, and right Pulvinar + LGN.

For each pair of ROIs located in the same hemisphere, we estimated possible white matter tracts that represent the anatomical connections between them. Tractography was constrained by the white matter mask, and a union mask of two ROIs was used as a seed. For each pair, we included only the fibers that traverse both ROIs. Tractography was performed using mrTrix software and the streamtrack command. For each set of ROIs, the algorithm discovered a maximum of 10,000 fibers with 1,000,000 trials. To improve the accuracy of our results, we used ensemble tractography [38], which performs the same tracking procedure but changes the lmax value and the minimum curvature value. We used four different lmax values l = [4 6 8] and five curvature values c = [0.25 0.5 1 2 4]. Finally, the obtained fibers were merged into one final fiber bundle. 

Obtained tracts were validated with LiFE software [39,40].The algorithm predicts the diffusion signal using the orientation of the fascicles present in obtained connections and compares it to the acquired MR data. The difference between the two is used to calculate prediction error. For each voxel, a weight is assigned that describes how each fascicle contributes toward predicting the diffusion model, with 0 signifying maximum error and 1 signifying no error. Fascicles with zero-weights were discarded from the analysis. This procedure was applied to all tracts.

#### 3.4.3. Mapping the Pulvinar and LGN ROIs from an Atlas 

Pulvinar and LGN ROIs in the intact hemisphere were mapped from the MNI atlas created as a part of the Natural Scenes Dataset [24]. To do that, we used a non-linear warping technique implemented in ANTs [41]. IB’s anatomical T1-weighted image was aligned to a standard anatomical template, the MNI atlas, using ANTs [42,43]. After finding the necessary transformation matrix and warp-field, we used its reverse form to map the ROIs to IB’s anatomical space.

## 4. Discussion

Despite missing the left eye (microphthalmos eye) and a massive lesion in the left hemisphere, which consists of a complete lack of thalamo-cortical connections of the left hemisphere, Patient IB shows remarkable residual visual capacities. The computerized perimetry indicated that light detection in the right hemifield was almost completely preserved up to 30° along the horizontal axis. At higher eccentricities, target detection was slightly and sparsely impaired only in the lower right visual field. Such a distortion is consistent with the observed difference between contrast thresholds for orientation discrimination. 

Our data indicate that the cortical reorganization in patient IB mediates residual contrast analysis to serve not only for the basic detection but also to discriminate between the stimulus properties such as motion and orientation. The retinotopic representation of the central full field in contralateral hemispheres supports the existence of normal residual vision in Patient IB. 

This raises several questions: What neural pathway mediates vision in the lesioned hemisphere? Where is the input to the left V1 coming from? All the visual information received from the right eye does not cross over to the left side through OC, as shown by our tractography results (Figure 4A). However, our data show reorganization of the thalamus in the intact hemisphere, given that it responds well to both the ipsi- and contra- lateral visual field.

Abnormal innervation of the optic track has been observed in many pathologies, such as albinism, congenital achiasma, microphthalmia, and more recently, FHONDA patients [6,44,45]. In all these pathologies, the fibers have an atypical decussation at chiasma, resulting in LGN and cortex receiving information from the entire visual field. However, in all these pathologies, the re-routing of visual pathways is very different from that which we observed in IB and other congenital lesion patients [18,22].

Only in the light of tractography results is it possible to hypothesize the putative input pathway associated with the right visual field. Primate studies suggest that the SC is a subcortical relay of retinal information to extrastriate visual cortex. Ascending SC projections pass through pulvinar and LGN on their way to cortex [46].

In both hemispheres, we find surprisingly good mapping of areas in the far peripheral visual field, possibly including the area prostriata. Previously, it was reported that mapping the area prostriata (located at the fundus of calcarine sulcus) requires retinotopic data that extends to 60 degrees of periphery [22]. Here, we obtain good fits with stimulus that extends only to 20 deg. Strong and reliable responses in the area prostriata might suggest that it could play a role in IB’s cortical organization especially with its connections being retinotopically organized [47].

The pulvinar/LGN to hMT+ tract in the intact hemisphere survives the strict analysis method that we implemented for tractography. We know from the literature that this tract is prominent at birth and is heavily pruned during development to practically disappear in adulthood [48,49,50,51,52]. It is possible that this tract might be involved in transferring some left visual field information to the intact hemisphere. 

In human infants, a complex network of higher visual associative areas is still not well established by 7 weeks of age [53]. In the absence of direct optic radiation innervating to V1 in damaged hemisphere, the feed-forward connections from V1 to hMT+ may be abnormal. This could lead to the strengthening of this extrastriate visual pathway in patients [8].Studies on blind-sight subjects corroborate this assumption [48]. Blindsight patients are characteristically unaware of stimuli presented in the scotoma, but they can perform above chance level in forced-choice tasks, even though there is damage in the corresponding V1 locations [7,8,54].Blindsight patients show a clear capacity of processing ‘dorsal’ visual stream properties, such as luminance, contrast, or flicker and motion. This leads to the idea that a direct projection from subcortical structures to hMT+ might sustain residual visual functions [8]. In case of a blindsight patient G.Y., whose left V1 is entirely missing, a strong ipsilateral connection is linking LGN and hMT+ [45]. The direct pathway connecting the LGN to hMT+ has been recently linked to account for developing blindsight after a lesion to primary visual cortex in adulthood [8]. In the case of Patient IB, the lesioned hemisphere seems to have retained a strong connection between SC and hMT+, presumably bypassing the pulvinar and LGN. This is supported by tractography results showing a strong SC to hMT+ tract in the lesioned hemisphere. However, while the blindsight patients of Ajina et al. (2015) [8] showed dense scotomas given the V1 lesions, IB has practically complete vision with small deficits in contrast threshold. This suggests that as long as V1 has a proper retinotopic organization, it can mediate good and conscious vision. In the absence of V1 activity, the awareness or consciousness of the visual stimulus would be greatly impaired. This result is consistent with the perception of another congenital patient with complete lesion of optic radiation and a good retinotopic representation of V1 [22].In this patient, the visual input to V1 is via aberrant pathways involving putative hMT+; nevertheless, the vision of the patient was nearly complete and always conscious.

It is interesting to compare our results with a case study reported by Muckli et al. [18].They report a 10-year-old patient (AH) who lacks the entire right cortical hemisphere and most of her right eye (microphthalmia). The patient’s spared hemisphere has representation of both the contralateral (right) visual hemifield and the ipsilateral (left) visual hemifield. They rightfully conclude that the retinal ganglion cells changed their predetermined crossing pattern in the optic chiasm to innervate the ipsilateral LGN. As in our subject IB, LGN of AH represents both left and right visual field. In the patient of Muckli et al. (2009) [18], the intact hemisphere contains islands of retinotopic representation of the ipsilateral visual field, while our patient has a complete and normal representation in both hemispheres. The lesion in Patient IB is less pronounced, sparing the primary and some associative visual cortex in the damaged hemisphere. Hence, in IB, the primary areas can be used to generate visual maps with rerouted visual information, and this can also occur when the input is primarily from SC instead of from the thalamus. Both patients show that a very strong and reliable mechanism can guide axonal growth and innervation, allowing functional compensation of the anatomical deficit. Clear indication from an animal model with lesions performed at embryonic age demonstrates that neuronal mapping formation is governed by the correlation of electric discharge: correlated waves of activity at the retinal level generate the segregation of eye input at LGN and at the thalamic level generate ordered retinotopic or somatotopic cortical maps [55,56,57,58]. Our study demonstrates that in the pathological brain, abnormal thalamic projections can be formed to obtain a normal retinotopic map in the visual cortex, as retinotopic organization is a stringent requirement for a complete and conscious vision. Surprisingly, to innervate V1, the abnormal connection may connect first the thalamic nucleus to contralateral SC and then to the extrastriate cortical area hMT+ to finally reach V1 of the lesioned hemisphere.

## Data Availability

Akshatha Bhat, Jan W. Kurzawski, Giovanni Anobile, Francesca Tinelli, Laura Biagi, & Maria Concetta Morrone. (2022). Normal Retinotopy in Primary Visual Cortex in a Congenital Complete Unilateral Lesion of Lateral Geniculate Nucleus in Human: A Case Study (Data set). Zenodo. https://doi.org/10.5281/zenodo.5873773 (accessed on 30 December 2021).

## References

[1] Hubel D.H., Wiesel T.N. (1962). Receptive Fields, Binocular Interaction and Functional Architecture in the Cat’s Visual Cortex. J. Physiol..

[2] Wiesel T.N., Hubel D.H. (1965). EXTENT OF RECOVERY FROM THE EFFECTS OF VISUAL DEPRIVATION IN KITTENS. J. Neurophysiol..

[3] Petros T.J., Rebsam A., Mason C.A. (2008). Retinal Axon Growth at the Optic Chiasm: To Cross or Not to Cross. Annu. Rev. Neurosci..

[4] Mcketton L., Kelly K.R., Schneider K.A. (2014). Abnormal Lateral Geniculate Nucleus and Optic Chiasm in Human Albinism: Abnormal Visual System Morphology in Albinism. J. Comp. Neurol..

[5] Puzniak R.J., Ahmadi K., Kaufmann J., Gouws A., Morland A.B., Pestilli F., Hoffmann M.B. (2019). Quantifying Nerve Decussation Abnormalities in the Optic Chiasm. NeuroImage Clin..

[6] Ahmadi K., Fracasso A., van Dijk J.A., Kruijt C., van Genderen M., Dumoulin S.O., Hoffmann M.B. (2019). Altered Organization of the Visual Cortex in FHONDA Syndrome. NeuroImage.

[7] Tinelli F., Cicchini G.M., Arrighi R., Tosetti M., Cioni G., Morrone M.C. (2013). Blindsight in Children with Congenital and Acquired Cerebral Lesions. Cortex.

[8] Ajina S., Pestilli F., Rokem A., Kennard C., Bridge H. (2015). Human Blindsight Is Mediated by an Intact Geniculo-Extrastriate Pathway. eLife.

[9] Papanikolaou A., Keliris G.A., Papageorgiou T.D., Shao Y., Krapp E., Papageorgiou E., Stingl K., Bruckmann A., Schiefer U., Logothetis N.K. (2014). Population Receptive Field Analysis of the Primary Visual Cortex Complements Perimetry in Patients with Homonymous Visual Field Defects. Proc. Natl. Acad. Sci. USA.

[10] Wandell B.A., Smirnakis S.M. (2009). Plasticity and Stability of Visual Field Maps in Adult Primary Visual Cortex. Nat. Rev. Neurosci..

[11] Teuber H.-L., Porter R., Fitzsimons D.W. (2008). Recovery of Function After Brain Injury in Man. Novartis Foundation Symposia.

[12] Perenin M.T. (1978). Visual Function within the Hemianopic Field Following Early Cerebral Hemidecortication in Man—II. Pattern Discrimination. Neuropsychologia.

[13] Knyazeva M.G., Maeder P., Kiper D.C., Deonna T., Innocenti G.M. (2002). Vision After *Early-Onset* Lesions of the Occipital Cortex: II. Physiological Studies. Neural Plast..

[14] Werth R. (2006). Visual Functions without the Occipital Lobe or after Cerebral Hemispherectomy in Infancy. Eur. J. Neurosci..

[15] Braddick O., Atkinson J., Hood B., Harkness W., Jackson G., Vargha-Khademt F. (1992). Possible Blindsight in Infants Lacking One Cerebral Hemisphere. Nature.

[16] Gómez-Pinilla F., Villablanca J.R., Sonnier B.J., Levine M.S. (1986). Reorganization of Pericruciate Cortical Projections to the Spinal Cord and Dorsal Column Nuclei after Neonatal or Adult Cerebral Hemispherectomy in Cats. Brain Res..

[17] Carr L.J., Harrison L.M., Evans A.L., Stephens J.A. (1993). Patterns of Central Motor Reorganization in Hemiplegic Cerebral Palsy. Brain.

[18] Muckli L., Naumer M.J., Singer W. (2009). Bilateral Visual Field Maps in a Patient with Only One Hemisphere. Proc. Natl. Acad. Sci. USA.

[19] Chambers T.M., Agopian A.J., Lewis R.A., Langlois P.H., Danysh H.E., Weber K.A., Shaw G.M., Mitchell L.E., Lupo P.J. (2018). Epidemiology of Anophthalmia and Microphthalmia: Prevalence and Patterns in Texas, 1999–2009. Am. J. Med. Genet. A..

[20] Hoffmann M.B., Dumoulin S.O. (2015). Congenital Visual Pathway Abnormalities: A Window onto Cortical Stability and Plasticity. Trends Neurosci..

[21] Neveu M.M., Holder G.E., Ragge N.K., Sloper J.J., Collin J.R.O., Jeffery G. (2006). Early Midline Interactions Are Important in Mouse Optic Chiasm Formation but Are Not Critical in Man: A Significant Distinction between Man and Mouse. Eur. J. Neurosci..

[22] Mikellidou K., Kurzawski J.W., Frijia F., Montanaro D., Greco V., Burr D.C., Morrone M.C. (2017). Area Prostriata in the Human Brain. Curr. Biol..

[23] Engel S. (1997). Retinotopic Organization in Human Visual Cortex and the Spatial Precision of Functional MRI. Cereb. Cortex.

[24] Allen E.J., St-Yves G., Wu Y., Breedlove J.L., Prince J.S., Dowdle L.T., Nau M., Caron B., Pestilli F., Charest I. (2021). A Massive 7T FMRI Dataset to Bridge Cognitive Neuroscience and Artificial Intelligence. Nat. Neurosci..

[25] Arcaro M.J., Pinsk M.A., Kastner S. (2015). The Anatomical and Functional Organization of the Human Visual Pulvinar. J. Neurosci..

[26] Brainard D.H. (1997). The Psychophysics Toolbox. Spat. Vis..

[27] Cox R.W. (1996). AFNI: Software for Analysis and Visualization of Functional Magnetic Resonance Neuroimages. Comput. Biomed. Res..

[28] Saad Z.S., Glen D.R., Chen G., Beauchamp M.S., Desai R., Cox R.W. (2009). A New Method for Improving Functional-to-Structural MRI Alignment Using Local Pearson Correlation. NeuroImage.

[29] Kay K.N., Rokem A., Winawer J., Dougherty R.F., Wandell B.A. (2013). GLMdenoise: A Fast, Automated Technique for Denoising Task-Based FMRI Data. Front. Neurosci..

[30] Dumoulin S.O., Wandell B.A. (2008). Population Receptive Field Estimates in Human Visual Cortex. NeuroImage.

[31] Dumoulin S.O., Harvey B.M. (2012). Reconstructing Human Population Receptive Field Properties. J. Vis..

[32] Friston K.J., Fletcher P., Josephs O., Holmes A., Rugg M.D., Turner R. (1998). Event-Related FMRI: Characterizing Differential Responses. NeuroImage.

[33] Himmelberg M.M., Kurzawski J.W., Benson N.C., Pelli D.G., Carrasco M., Winawer J. (2021). Cross-Dataset Reproducibility of Human Retinotopic Maps. NeuroImage.

[34] Wandell B.A., Dumoulin S.O., Brewer A.A. (2007). Visual Field Maps in Human Cortex. Neuron.

[35] Worsley K.J., Liao C.H., Aston J., Petre V., Duncan G.H., Morales F., Evans A.C. (2002). A General Statistical Analysis for FMRI Data. NeuroImage.

[36] Tournier J.-D., Calamante F., Gadian D.G., Connelly A. (2004). Direct Estimation of the Fiber Orientation Density Function from Diffusion-Weighted MRI Data Using Spherical Deconvolution. NeuroImage.

[37] Tournier J.-D., Calamante F., Connelly A. (2012). MRtrix: Diffusion Tractography in Crossing Fiber Regions. Int. J. Imaging Syst. Technol..

[38] Takemura H., Caiafa C.F., Wandell B.A., Pestilli F. (2016). Ensemble Tractography. PLOS Comput. Biol..

[39] Pestilli F., Yeatman J.D., Rokem A., Kay K.N., Wandell B.A. (2014). Evaluation and Statistical Inference for Human Connectomes. Nat. Methods.

[40] Caiafa C.F., Pestilli F. (2017). Multidimensional Encoding of Brain Connectomes. Sci. Rep..

[41] Avants B.B., Tustison N.J., Song G., Cook P.A., Klein A., Gee J.C. (2011). A Reproducible Evaluation of ANTs Similarity Metric Performance in Brain Image Registration. NeuroImage.

[42] Collins D.L., Holmes C.J., Peters T.M., Evans A.C. (1995). Automatic 3-D Model-based Neuroanatomical Segmentation. Hum. Brain Mapp..

[43] Mazziotta J., Toga A., Evans A., Fox P., Lancaster J., Zilles K., Woods R., Paus T., Simpson G., Pike B. (2001). A Probabilistic Atlas and Reference System for the Human Brain: International Consortium for Brain Mapping (ICBM). Philos. Trans. R. Soc. B Biol. Sci..

[44] Apkarian P., Bour L.J., Barth P.G., Wenniger-prick L., Verbeeten B. (1995). Non-Decussating Retinal-Fugal Fibre Syndrome: An Inborn Achiasmatic Malformation Associated with Visuotopic Misrouting, Visual Evoked Potential Ipsilateral Asymmetry and Nystagmus. Brain.

[45] Bridge H., Thomas O., Jbabdi S., Cowey A. (2008). Changes in Connectivity after Visual Cortical Brain Damage Underlie Altered Visual Function. Brain.

[46] Lyon D.C., Nassi J.J., Callaway E.M. (2010). A Disynaptic Relay from Superior Colliculus to Dorsal Stream Visual Cortex in Macaque Monkey. Neuron.

[47] Kurzawski J.W., Mikellidou K., Morrone M.C., Pestilli F. (2020). The Visual White Matter Connecting Human Area Prostriata and the Thalamus Is Retinotopically Organized. Brain Struct. Funct..

[48] Bourne J.A., Morrone M.C. (2017). Plasticity of Visual Pathways and Function in the Developing Brain: Is the Pulvinar a Crucial Player?. Front. Syst. Neurosci..

[49] Nakagawa S., Tanaka S. (1984). Retinal Projections to the Pulvinar Nucleus of the Macaque Monkey: A Re-Investigation Using Autoradiography. Exp. Brain Res..

[50] Perry V.H., Oehler R., Cowey A. (1984). Retinal Ganglion Cells That Project to the Dorsal Lateral Geniculate Nucleus in the Macaque Monkey. Neuroscience.

[51] Warner C.E., Kwan W.C., Bourne J.A. (2012). The Early Maturation of Visual Cortical Area MT Is Dependent on Input from the Retinorecipient Medial Portion of the Inferior Pulvinar. J. Neurosci..

[52] Warner C.E., Kwan W.C., Wright D., Johnston L.A., Egan G.F., Bourne J.A. (2015). Preservation of Vision by the Pulvinar Following Early-Life Primary Visual Cortex Lesions. Curr. Biol..

[53] Biagi L., Crespi S.A., Tosetti M., Morrone M.C. (2015). BOLD Response Selective to Flow-Motion in Very Young Infants. PLOS Biol..

[54] Tamietto M., Morrone M.C. (2016). Visual Plasticity: Blindsight Bridges Anatomy and Function in the Visual System. Curr. Biol..

[55] Antón-Bolaños N., Sempere-Ferràndez A., Guillamón-Vivancos T., Martini F.J., Pérez-Saiz L., Gezelius H., Filipchuk A., Valdeolmillos M., López-Bendito G. (2019). Prenatal Activity from Thalamic Neurons Governs the Emergence of Functional Cortical Maps in Mice. Science.

[56] Galli L., Maffei L. (1988). Spontaneous Impulse Activity of Rat Retinal Ganglion Cells in Prenatal Life. Science.

[57] Meister M., Wong R.L., Baylor D.A., Shatz C.J. (1991). Action the May.

[58] Sharma J., Angelucci A., Sur M. (2000). Induction of Visual Orientation Modules in Auditory Cortex. Nature.

